# One-Step Biosynthesis of α-Keto-γ-Methylthiobutyric Acid from L-Methionine by an *Escherichia coli* Whole-Cell Biocatalyst Expressing an Engineered L-Amino Acid Deaminase from *Proteus vulgaris*

**DOI:** 10.1371/journal.pone.0114291

**Published:** 2014-12-22

**Authors:** Gazi Sakir Hossain, Jianghua Li, Hyun-dong Shin, Guocheng Du, Miao Wang, Long Liu, Jian Chen

**Affiliations:** 1 Key Laboratory of Carbohydrate Chemistry and Biotechnology, Ministry of Education, Jiangnan University, Wuxi, China; 2 Key Laboratory of Industrial Biotechnology, Ministry of Education, Jiangnan University, Wuxi, China; 3 Synergetic Innovation Center Of Food Safety and Nutrition, Wuxi, China; 4 School of Chemical and Biomolecular Engineeirng, Georgia Institute of Technology, Atlanta, Georgia, United States of America; 5 School of Food Science and Technology, Jiangnan University, Wuxi, China; Consejo Superior de Investigaciones Cientificas, Spain

## Abstract

α-Keto-γ-methylthiobutyric acid (KMTB), a keto derivative of l-methionine, has great potential for use as an alternative to l-methionine in the poultry industry and as an anti-cancer drug. This study developed an environment friendly process for KMTB production from l-methionine by an *Escherichia coli* whole-cell biocatalyst expressing an engineered l-amino acid deaminase (l-AAD) from *Proteus vulgaris*. We first overexpressed the *P. vulgaris* l-AAD in *E. coli* BL21 (DE3) and further optimized the whole-cell transformation process. The maximal molar conversion ratio of l-methionine to KMTB was 71.2% (mol/mol) under the optimal conditions (70 g/L l-methionine, 20 g/L whole-cell biocatalyst, 5 mM CaCl_2_, 40°C, 50 mM Tris-HCl [pH 8.0]). Then, error-prone polymerase chain reaction was used to construct *P. vulgaris* l-AAD mutant libraries. Among approximately 10^4^ mutants, two mutants bearing lysine 104 to arginine and alanine 337 to serine substitutions showed 82.2% and 80.8% molar conversion ratios, respectively. Furthermore, the combination of these mutations enhanced the catalytic activity and molar conversion ratio by 1.3-fold and up to 91.4% with a KMTB concentration of 63.6 g/L. Finally, the effect of immobilization on whole-cell transformation was examined, and the immobilized whole-cell biocatalyst with Ca^2+^ alginate increased reusability by 41.3% compared to that of free cell production. Compared with the traditional multi-step chemical synthesis, our one-step biocatalytic production of KMTB has an advantage in terms of environmental pollution and thus has great potential for industrial KMTB production.

## Introduction

α-Keto-γ-methylthiobutyric acid (KMTB) is a keto form of the sulfur-based amino acid methionine. Methionine is essential to human and poultry health and cellular function, and the body must obtain this vital substance through dietary means. Because the free form of l-methionine is promptly decomposed by the bacterial flora of the intestine, only a very small portion of l-methionine circulates in the blood. However, the modification of l-methionine to its corresponding keto acid improves the bioavailability of methionine. In addition, this product is of increasing interest in the pharmaceutical industry: two-thirds of all tumors are methionine dependent, and these types of tumor cells are deficient in KMTB and are therefore unable to undergo apoptosis [1], [2], [3]. Thus, KMTB is an indirect inhibitor of cell growth in culture via the methional metabolic pathway. In addition, the testing of KMTB in colon cancer has demonstrated that it is a safe, well-tolerated therapy capable of limiting tumor growth [4]. KMTB has also been evaluated as a methionine supplement in livestock feed without any reported toxicity [5], and it has been used in the treatment of uremic patients.

Currently, KMTB is produced via chemical synthesis, which is not only a multi-step process starting from ethyloxylyl chloride but also uses harsh chemicals and produces toxic wastes [6], [7]. Therefore, considerable interest has been generated in the development of ecofriendly technology for the production of KMTB from l-methionine through biocatalysis. The enzymatic synthesis of KMTB from d-methionine using the d-amino acid oxidase (d-AAO; EC 1.4.3.3) of *Trigonopsis variabilis* CBS 409 [8] has been described, but this reaction produces hydrogen peroxide. As a result, catalase must be used with d-AAO to produce α-keto acid, which ultimately increases the cost of production. Moreover, the nascent α-keto acid is non-enzymatically converted to the corresponding carboxylic acid in the presence of hydrogen peroxide. Thus, this reactive side product is extraordinarily disadvantageous for KMTB production by d-AAO. Additionally, hydrogen peroxide has a strong denaturing effect on proteins and, therefore, influences the operational stability of d-AAO. The industrial application of KMTB depends on the cost of the catalyst as well as the cost of the substrate. Compared to d-methionine, l-methionine is much cheaper and economically viable for industrial KMTB production. Therefore, this work aimed to design and develop a biocatalytic process for the deamination of l-methionine into KMTB using an immobilized whole-cell biocatalyst.

First, the full-length l-amino acid deaminase (l-AAD) from *Proteus vulgaris* was overexpressed in *Escherichia coli* BL21 (DE3), and the biotransformation process was optimized to achieve maximal bioconversion of l-methionine to KMTB. Then, two positive l-AAD mutants were obtained via error-prone polymerase chain reaction (er-PCR), and additional combinational site-directed mutation was carried out to enhance the catalytic performance of the biocatalyst. Finally, the influence of whole-cell biocatalyst immobilization on l-methionine transformation was investigated. This study achieved the effective biotransformation of l-methionine to KMTB via protein engineering of l-AAD from *P. vulgaris*, and the strategy described herein may be useful for the biosynthesis of the other α-keto acids via biotransformation.

## Method and Materials

### Strains, vectors, and materials

The bacterial strains, plasmids, and primers used in this study are listed in Table 1. An EZ-10 Spin Column Plasmid MiniPreps kit, DNA purification kit, restriction enzymes, and T4 DNA ligase were purchased from TaKaRa (Dalian, China). Other chemicals as well as primer synthesis and DNA sequencing were provided by Shanghai Sangon Biological Engineering Technology and Services Co. Ltd. (Shanghai, China). Ampicillin and chloramphenicol were purchased from Amresco (Solon, OH, USA), and isopropyl-β-d-1-thiogalactopyranoside (IPTG) was purchased from Merck (Darmstadt, Germany). Sodium salt of KMTB was purchased from Sigma-Aldrich (St. Louis, MO, USA). All other chemicals were commercially available reagents of analytical grade. *E. coli* seed cultures were initiated in Luria-Bertani (LB) medium, and the production of l-AAD and the growth of whole-cell biocatalysts were performed in Terrific Broth.

**Table 1:** Oligonucleotide primers, plasmids and strains used in this study.

Primers/Plasmids/ Strains	Nucleotide sequence (5′→3′)^a^/description	Restriction enzyme/sources
**Primers:**		
LAAD_F	CGCGGATCCATGGCAATAAGTAGAAGAAAATTTA	*Bam*HI
LAAD_R	CCGCTCGAGTTAGAAACGATACAGACTAAATGGT	*Xho*I
A337S_F	TATTATCATTACCTGATTTCCCTGTGCATA	
A337S_R	TGGCAGATATTTATAGCCATAAGTGAAGG	
**Plasmids:**		
pET-20b (+)		Invitrogen, Carlsbad, CA
**Strains:**		
*E. coli* BL21(DE3)		Invitrogen, Carlsbad, CA
*P. vulgaris*		Japan Collection of Microorganism Saitama, Japan

a Nucleotides underlined correspond to the restriction enzyme site and codon chosen for mutation.

### l-AAD plasmid construction and transformation

*P. vulgaris* was purchased from the Japan Collection of Microorganisms (Institute of Physical and Chemical Research, Wako, Saitama, Japan). The genomic DNA was extracted with a Genomic DNA Purification Kit (Thermo Scientific, Waltham, MA, USA). For cloning into pET-20b (+), full-length *l-aad* was amplified with PCR using a gene-specific forward primer (LAAD_F) and reverse primer (LAAD_R). The PCR product was digested with *Bam*HI and *Xho*I, purified, and ligated into pET-20b (+) for expression in *E. coli*. The recombinant constructs were confirmed through restriction analysis and verified with DNA sequencing.

### Membrane preparation, cell density determination, and enzyme activity assays

The cultures of control and recombinant strains were centrifuged at 8,000×*g* for 10 min, and the supernatants were used to check the extracellular enzyme activity. The cell pellet from 500 mL of culture was resuspended in 20 mL of lysis buffer A consisting of 50 mM Tris-HCl buffer (pH 8.0), 1 mM phenylmethanesulfonyl fluoride, 1 mM ethylenediaminetetraacetic acid, and 1 mM dithiothreitol and ultrasonicated for 40 min on ice (cycles of 1 s sonication and 2 s pause) using a Vibra-Cell sonicator (Sonics, Newtown, CT, USA). The sonicate was centrifuged at low speed (4,000×*g*, 8 min, 4°C) to remove unbroken cells, and the resulting supernatant was centrifuged at high speed (110, 000×*g*, 1.5 h, 4°C) to obtain the pellets of the membranes [9]. The supernatant (designated the cytosolic fraction) was removed and stored at −80°C. The membrane pellets were washed twice with Tris-buffered saline, resuspended in 1 mL of Tris-HCl buffer containing 20% glycerol, pH 8.0, frozen in liquid nitrogen, and stored at −80°C. The cell membrane and cytosolic fractions were subsequently analyzed with sodium dodecyl sulfate-polyacrylamide gel electrophoresis and assayed for l-AAD activity.

For the assay of l-AAD activity, samples were incubated at 40°C with 468 mM l-methionine and 50.0 mM Tris-HCl buffer (pH 8.0) in a final volume of 2.0 mL. The reaction was stopped after 60 min by adding 1.0 mL of 20% trichloroacetic acid, and the concentration of KMTB was measured using high-performance liquid chromatography (HPLC). One unit of l-AAD was defined as the amount of membrane protein that generated 1 µM of KMTB per minute. To determine the dry cell weight (DCW), we centrifuged 100 mL of culture broth at 10,000×*g* for 10 min. The pellet was washed with 0.9% (w/v) NaCl, centrifuged at 10,000×*g* for 10 min, and dried to constancy at 105°C. The total protein concentration was measured using a BCA assay kit (Tiangen, China), and bovine serum albumin was used as a standard.

### Preparation of the whole-cell biocatalyst

For preparation of seed cultures, recombinant *E. coli* cells were grown in LB medium containing ampicillin (100 mg/L; *E. coli*) for 12 h at 37°C on a rotary shaker (200 rpm). The seed cultures (1%, v/v) were then inoculated into Terrific Broth in a 3-L vessel (BioFlo 115, New Brunswick Scientific Co., Edison, NJ, USA) with a working volume of 1.8 L. When the optical density at 600 nm of the cultures reached 0.6, which was determined in pilot experiments to be the optimal time for l-AAD induction, IPTG was added to a final concentration of 0.4 mM. The agitation speed, aeration rate, and temperature were maintained at 400 rpm, 1.0 vvm, and 25°C, respectively, to avoid the formation of inactive inclusion bodies. After 5 h of induction, the cells were harvested via centrifugation at 8,000×*g* for 10 min at 4°C, and the pellets were washed twice with 20 mM Tris-HCl buffer (pH 8.0). The cell pellet was then resuspended in the same buffer and maintained at 4°C for further studies. The biomass concentrations were measured spectrophotometrically (UV-2450 PC, Shimadzu Co., Kyoto, Japan) and converted to DCW using the following equation: DCW (g/L)  =  (0.4442× optical density at 600 nm) −0.02.

### Assay of whole-cell biocatalytic activity

Erlenmeyer flasks (100 mL) were used in all reactions in which KMTB was produced. The reaction mixture (20 mL) contained 50 mM Tris-HCl buffer (pH 8.0) and 5 mM MgCl_2_. For the assay of whole-cell biocatalytic activity, 20.0 g/L whole-cell biocatalyst and 70 g/L l-methionine were incubated on a rotary shaker at 220 rpm and 40°C for 24 h. The reaction was stopped by centrifugation at 8,000×*g* for 10 min at 4°C, and the supernatant was recovered for HPLC measurement of KMTB, as described below.

### Optimization of temperature, pH, agitation speed, aeration rate, and biocatalyst and substrate concentrations

Optimization was performed with 20 mL of reaction mixture in a 100-mL Erlenmeyer flask for all variables except aeration rate and agitation speed, which were optimized using a 3-L bioreactor containing 1.4 L of reaction mixture. With the exception of the variables indicated below, reactions were performed using the standard whole-cell biocatalytic reaction conditions described above. To optimize temperature, the reaction was performed at pH 8.0 and 220 rpm with temperatures varying between 20°C and 60°C. For pH optimization, the conditions were 40°C, 220 rpm, and Na_2_HPO_4_–KH_2_PO_4_ buffer (pH 5–9). For optimization of agitation speed and aeration rate in a 3-L fermenter, the reaction conditions were 40°C, pH 8.0, agitation speed between 200 and 600 rpm, and aeration rate between 0.5 and 2.5 vvm. For optimization of the whole-cell biocatalyst concentration, the conditions were 40°C, 220 rpm, pH 8.0, and cell concentration between 5 and 40 g/L. For optimization of substrate concentration, the conditions were 40°C, 220 rpm, pH 8.0, and l-methionine concentration between 5 and 70 g/L. The reactions were stopped by centrifugation at 8,000×*g* for 10 min, and the supernatant was recovered for HPLC measurement of KMTB.

### Analysis of KMTB concentration using HPLC

The amount of KMTB in the reaction mixture was determined with HPLC (Agilent 1200 series, Santa Clara, CA, USA) using a Phenomenex 5 µm Kromasil C8 column (150 mm and 4.6 mm; Phenomenex, Torrance, CA, USA) at a flow rate of 1 mL min^−1^ and a fixed wavelength of 210 nm in a mobile phase consisting of 15 mM KH_2_PO_4_, 30% methanol, and 10 mM tetrabutylammonium hydrogen sulfate, pH 6.5. The column temperature was maintained at 35°C, and the injection volume was 10 µL. KMTB was detected with a diode detector at a wavelength of 210 nm.

### Determination of substrate specificity

Substrate specificity was determined by dissolving 10 mM of the amino acids l-alanine, l-arginine, l-asparagine, l-glutamic acid, l-glutamine, l-histidine, l-leucine, l-lysine, l-methionine, l-phenylalanine, l-proline, l-threonine, and l-tryptophan and 5 mM of l-tyrosine in 500 µL of 50 mM Tris-HCl buffer (pH 8.0). The final solution was incubated with 100 µg of protein for 60 min at 40°C. The reaction was blocked with 500 µL of 20% trichloroacetic acid (w/v), and the keto acid was determined as described below. Reactions were repeated twice with each substrate, and two reactions using an assay solution with water (18.2 Ω. cm) as the substrate were included as control reactions.

### Effect of metal ions on enzyme activity

To determine the effect of metal ions on enzyme activity, we measured l-AAD activity in 50 mM Tris-HCl buffer (pH 8.0) containing Na^+^, K^+^, Li^+^, Ba^2+^, Fe^2+^, Mg^2+^, Mn^2+^, Cu^2+^, Zn^2+^, Ca^2+^ Co^+^, Ni^+^, and Al^2+^ (5 mM).

### Determination of the kinetic parameters of the whole-cell biocatalyst containing l-AAD and its mutants

The whole-cell biocatalyst containing l-AAD was incubated with various concentrations of l-methionine substrate to determine its kinetic parameters. The amount of substrate converted into the product, KMTB, was measured with HPLC as described above. Initial velocities were determined by plotting the amount of KMTB as a function of time. The specific activity was defined as the grams of product formed per gram of biocatalyst per hour. The kinetic parameters *K*_m_ and *V*_max_ were determined with the Michaelis–Menten equation, *v* =  (*V*_max_[*S*])/(*K*_m_ + [*S*]), and Eadie-Hofstee plot, *v* = (−*K*_m_•*v*/[*S*]) + *V*_max_, where *V*_max_ is the maximum activity, *K*_m_ is the Michaelis constant, and [*S*] is substrate concentration. The theoretical molecular mass was calculated using the tool at http://web.expasy.org/compute_pi/.

### α-Keto acid analysis

As a carbonyl derivative, α-keto acid was detected using 2, 4-dinitrophenylhydrazine (DNP). DNP reacts with carbonyl groups to produce dinitro-phenylhydrazone, which is brownish-red. Briefly, 500 µL of 10 mM l-amino acid was mixed with 10 mg of whole-cell biocatalyst and incubated at 37°C for 60 min. The reaction was terminated by adding 450 µL of 20% trichloroacetic acid and kept at room temperature for 30 min. Next, 200 µL of 20 mM DNP was added, and the mixture was incubated at room temperature for 15 min. The reaction was terminated after the addition of 4 mL of 0.8 M NaOH and additional incubation for 15 min at room temperature. Finally, the mixture was centrifuged and the supernatant was used to measure the absorbance at 520 nm. A control reaction was used to subtract the background absorbance without l-amino acid.

### Random mutagenesis with er-PCR, mutant library construction, and screening

To perform er-PCR, we amplified l-*aad* from the recombinant plasmid pET-20b-LAAD. The reaction mixture for a 50- µL er-PCR sample contained 5 µL of 10× Mutazyme II reaction buffer, 1 µL of 40 mM deoxyribonucleotide triphosphate mix (200 µM each, final), 0.5 µL of primer mix (250 ng/ µL of each primer), 1 µL of Mutazyme II DNA polymerase (2.5 U/ µL), 1 µL of template, and 41.5 µL of water. The optimum amount of target DNA was used to produce a sufficient amount of mutants. After PCR, the products were purified and digested with *Bam*HI and *Xho*I and ligated with the pET20 b(+) plasmid to the corresponding site. The ligated products were then transformed to *E. coli* BL 21 (DE3) to produce the mutant library and screen the mutants. Variants from the mutant library were picked and cultured overnight in 0.4 mL of LB broth containing 0.1 g/L ampicillin in a microtube at 37°C. Seed broth was inoculated into Terrific Broth medium containing 0.1 g/L ampicillin at 37°C for 2 h and then induced by the addition of 0.2 mM IPTG at 30°C for 5 h. Biomass was collected via centrifugation and washed with 50 mM Tris-HCl buffer. Catalytic activity and bioconversion of l-methionine were measured as described above.

### Construction of double-mutant l-AAD

A double mutant was constructed through site-directed mutagenesis. First, the recombinant plasmid was amplified with PCR using mutagenic oligonucleotides (see Table 1) and the MutanBEST Kit (TaKaRa). Then, the amplified fragments were purified and isolated from 0.8% (w/v) agarose gels after electrophoresis. The fragment was blunted with Blunting Kination Enzyme Mix (TaKaRa). The blunt-end fragment was ligated with Ligation Solution I (TaKaRa). The competent cells of *E. coli* JM109 were then transformed by the reaction mixture. Selections of the transformants were performed on LB agar plates containing 50 µg/mL ampicillin. *l-AAD* in the transformant was confirmed with PCR and checked through sequencing, and the recombinant plasmids were finally transformed into competent cells of *E. coli* BL21 (DE3) for expression.

### Immobilization of the whole-cell biocatalyst

For immobilization in agar and 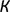-carrageenan, 0.4 g cells (DCW) were completely mixed with 20 mL of sterilized 4% (w/v) agar and 3% (w/v) 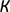-carrageenan at 45°C to 50°C. Agar containing cells was allowed to set at room temperature; when the cells were mixed with 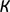-carrageenan, the mixture was placed at 4°C for 30 min before immersion in 0.3 mol/L KCl at 4°C for 4 h. The gel was cut in small cubes (0.3 cm×0.3 cm×0.3 cm) and washed with 50 mM Tris-HCl buffer (pH 8.0). The immobilized biocatalyst was stored at 4°C until use. For the immobilization in Ca^2+^ alginate, 0.4 g cells (DCW) were completely mixed with 20 mL of sterilized 2% (w/v) alginate sodium. Then, the mixture was added drop-wise from a syringe to a gently stirred solution of 200 mM CaCl_2_ to make beads with diameters between 2.5 and 3.0 mm. The resulting gel beads were hardened at room temperature for 2 h and kept at 4°C for 10 h. Finally, the beads were filtered, washed, and stored in 50 mM Tris-HCl buffer (pH 8.0) at 4°C until use. Freshly prepared beads were used for each experiment, and the entire cell immobilization procedure was performed under aseptic conditions. To determine the cell loading of the beads, we collected 30 gel beads in a test tube. Then, 9 mL of 50 mM sodium citrate solution was added to liquefied alginate matrix, and cells were released from the perforated membrane. The solution was diluted as required, and the absorbance at 420 nm was measured and converted to cell concentration (g-cell [dry cell weight]/L) by using a calibration curve.

### Statistical analysis

All experiments were performed at least three times, and the results are expressed as the mean ± standard deviation (n = 3). Data were analyzed using the Student's t test. *P* values less than 0.05 were considered statistically significant.

## Results and Discussion

### Cloning, expression, cellular localization, and substrate specificity of l-AAD from *P. vulgaris* in *E. coli*

**Figure 1:**
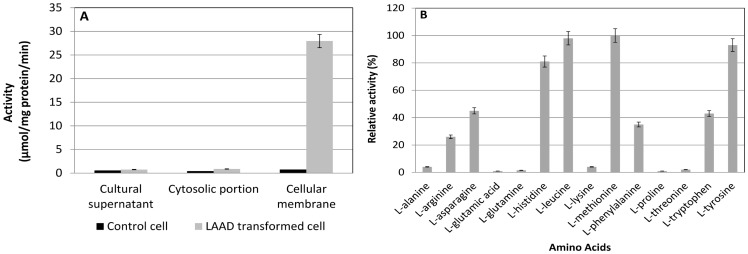
Cellular localization and substrate specificity. (A) Activity of l-AAD in the different cellular location; (B) Substrate specificity of l-AAD after heterologous expression in the *E. coli*. The highest activity of the l-AAD biocatalyst with l-methionine was defined as 100%.

The wild-type l-*aad* from *P. vulgaris* was cloned into pET-20b (+) expression vectors and sequenced. The recombinant plasmid was then transformed into *E. coli* BL21 (DE3) for l-AAD expression. l-AAD activity was then measured in the cytosolic fractions, membrane fractions, and culture supernatants after 5 h of induction with IPTG at 25°C (Fig. 1A). Recombinant l-AAD activity was found in the membrane fraction, and the result confirmed the localization of l-AAD of *P. vulgaris* in the membrane. The substrate specificities of the l-AAD biocatalyst for various amino acids were also examined, and l-AAD showed the highest specificity for l-methionine, followed by l-leucine and l-tyrosine (Fig. 1B). These results indicated that the heterologous expression in *E. coli* did not affect the substrate specificity of the enzyme [10]. We also compared the biotransformation capability of KMTB from l-methionine with another biocatalyst containing *P. mirabilis* l-AAD [11] and found that the biocatalyst containing *P. vulgaris* l-AAD had a higher l-methionine biotransformation efficiency (S1 Fig.).

Given these results, we proceeded with a biochemical analysis of the whole-cell biocatalytic activity of recombinant l-AAD–expressing *E. coli*. The l-AAO from other bacterial sources, such as, *Marinomonas mediterranea* and marine bacterium D2, display antimicrobial properties [12], [13]. To determine whether the expression of l-AAD of *P. vulgaris* affected the growth of *E. coli*, we examined the growth characteristics. The cell growth curve showed that the engineered l-AAD–expressing strains grew at a rate similar to that of non-transformed cells, suggesting that the overexpression of l-AAD from *P. vulgaris* in *E. coli* had little influence on cell growth. Because the functions of marine l-AAOs and *P. vulgaris* l-AAD are different, their effects on cell growth also differed. In addition, those l-AAOs produce hydrogen peroxide by which they exert their antimicrobial activity, but l-AADs from *Proteus* sp. oxidize l-amino acids without producing hydrogen peroxide [14].

### Biochemical characterization and process optimization for whole-cell biotransformation

**Figure 2:**
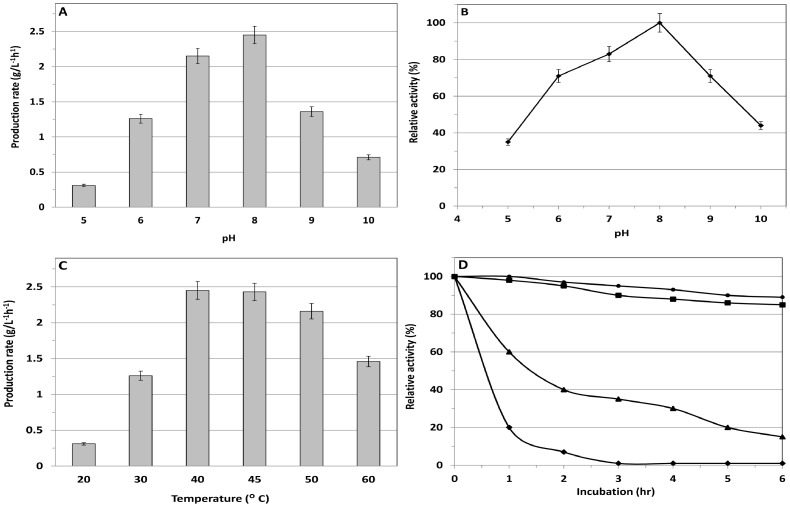
Influence of pH and temperature on whole cell catalytic activity. (A) influence of pH on the production rate; (B) stability of l-AAD in different pHs; (C) influence of temperature on the production rate and (D) stability of l-AAD in different temperatures (round sign (•)  = 30°C, square sign (▪)  = 40°C, triangle sign (▴)  = 50°C, and rectangle sign (♦)  = 60°C). The highest activity of the l-AAD containing biocatalyst (2.45 g/L^−1^h^−1^) was defined as 100%.

The optimal pH and pH stability of the whole-cell biocatalyst containing l-AAD were determined within a pH range of 3.0∼10.0. The results showed that l-AAD had an optimal pH of 8.0 (Fig. 2A) and retained more than 80% of its maximal activity between pH 7.5 and 8.5 (Fig. 2B). The optimal temperature of the whole-cell biocatalyst was determined within a range of 20∼60°C. The results showed that whole-cell biocatalytic activity increased with increasing temperature from 25°C to 40°C and decreased from 60°C (Fig. 2C). The maximum activity of the recombinant biocatalyst was observed at 40°C, and it maintained 70% of its maximal activity at 55°C (see Fig. 2C). The thermostability of the whole-cell biocatalyst was determined at 30°C, 40°C, 50°C, 60°C, and 70°C (Fig. 2D). The whole-cell biocatalyst was stable at 35∼40°C and became instable at temperatures above 50°C.

**Figure 3:**
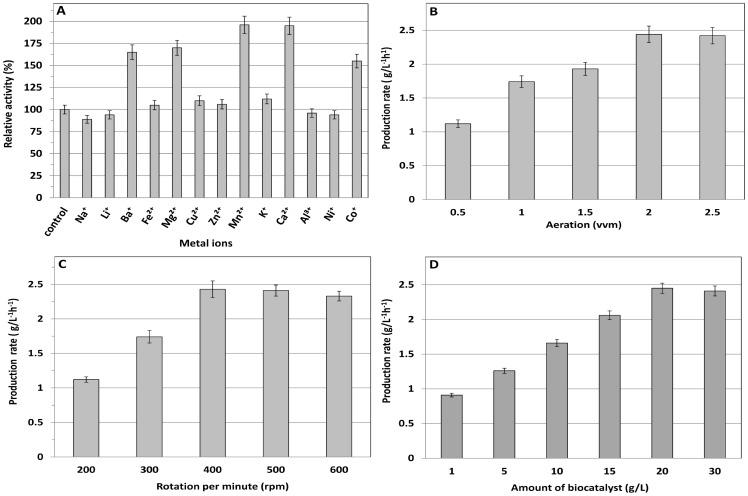
Influence of metal ions, aeration, agitation, and cell concentration on whole cell catalytic activity. (A) influence of different metal ions; (B) influence of aeration on the production rate; (C) influence of agitation on the production rate; and (D) effects of the biocatalyst concentration on the production rate.

When incubated with 5 mM of Ca^2+^, Mg^2+^, and Mn^2+^, the l-AAD biocatalyst stimulated deaminase activity, whereas incubation with Na^+^ resulted in a low inhibition effect (Fig. 3A). Because the *l-aad* gene sequence contains a region similar to that of the flavin adenine dinucleotide (FAD) binding site [10], we examined the effect of FAD concentration (5 to 50 µM) on enzyme activity. FAD concentration had no effect on the enzyme activity (data not shown), indicating that the external addition of FAD is not required for whole-cell biotransformation, which is similar to results reported for l-AAD (pm1) from *P.* mirabilis [11], [15]. From an industrial point of view, such whole-cell biocatalysis is advantageous because a costly cofactor will not be required for the biotransformation.

The effects of aeration and agitation were evaluated by incubating whole cells in a 3-L bioreactor. Aeration was assessed at a rate of 0.5 to 2.5 vvm, and 2.0 vvm was optimum for production (Fig. 3B). Agitation was checked from 200 to 600 rpm, and 400 rpm was optimal (Fig. 3C). Higher agitation did not improve the biotransformation rate. To determine the optimal biocatalyst content for biotransformation, we performed the reactions with cell concentrations ranging from 1 to 30 g-cell/L. KMTB production was optimal (49.2 g/L) at 20 g-cell/L and did not increase at higher cell concentrations (Fig. 3D). This finding suggested that high cell concentrations do not enhance KMTB production, perhaps owing to substrate and enzyme saturation. We next determined the optimal l-methionine concentration by testing concentrations between 5 and 70 g/L. KMTB production reached the highest level (2.4 g/L^−1^ h^−1^) at 70 g/L l-methionine, indicating that high substrate concentrations enhanced volumetric production by l-AAD.

### Improving the catalytic efficiency of the biocatalyst through direct evolution and site-directed mutation

**Figure 4:**
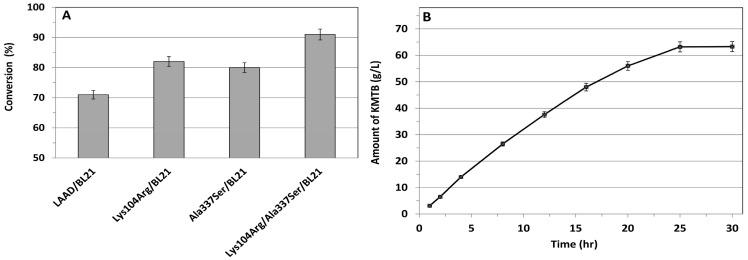
Result of mutagenesis on the molar conversion efficiency and time profile for product formation. (A) Conversion efficiency of different mutants from l-methionine to KMTB (mol/mol) and (B) Time profile for the production of KMTB (▪) from l-methionine by engineered double mutant l-AAD containing whole cell biocatalyst.

For industrial processes, directed evolution has become an influential tool in the production of variants that improve enzyme structure as well as function for specific purposes. Because the similarity of l-AAD to other l-AAOs for which crystal structures have been determined is very low (less than 20%), we could not predict the active site by developing homology modeling to improve biocatalytic efficiency. Therefore, random mutagenesis was used to improve the bioconversion efficiency of l-AAD in this study. Using er-PCR, we obtained two mutants with increased turnover of l-methionine to KMTB. The initial activities of these mutants were higher than that of l-AAD. Using sequencing analysis, we determined the mutations responsible for the increased activity and biotransformation efficiency. In the first point mutation, lysine 104 was replaced by arginine, which increased the bioconversion ratio from 71.2% to 82.2% (Fig. 4A). We found that the maximal reaction rate (*V*_max_) of the mutant l-AAD (Lys104Arg) was higher than that of the wild type (Table 2). Moreover, compared to that of the wild-type l-AAD, the *K*_m_ of the mutant also decreased, and as a result, *V*_max_/*K*_m_ increased (see Table 2, S2 Fig.).

**Table 2:** Apparent kinetic parameters of KMTB production using wild type and mutant l-AAD containing whole-cell biocatalysts.

Biocatalyst a	*K*_m_ (mM)	*V*_max_ (mM/g^−1^h^−1^)	*V*_max_/*K*_m_ (h^−1^)
LAAD/*E. coli* BL21 (DE3)	305±2.31	0.91±0.01	0.00298
Lys104Arg/*E. coli* BL21 (DE3)	264±2.07	1.05±0.02	0.00397
Ala337Ser/*E. coli* BL21 (DE3)	272±1.05	1.01±0.02	0.00368
Lys104Arg.Ala337Ser/*E. coli* BL21 (DE3)	238±1.08	1.14±0.04	0.00478

a The volume of each reaction mixture was 20 ml, and amount of biocatalyst in the reaction solutions was equal in quantity. All experiments were carried out at 40°C in 20 mL of 50 mM Tris–HCl (pH 8.0). *K_m_* values are expressed in terms of mM of substrate.

The kinetic results indicated that the affinity and catalytic efficiency of the mutant for l-methionine increased compared to that of the wild-type l-AAD. In the second point mutation, alanine 337 was replaced by serine, which increased the bioconversion ratio from 71.2% to 80.8% (see Fig. 4A). Similar to results with the previous mutation, the *K*_m_ value of the mutant l-AAD (Ala337Ser) was also decreased compared with that of the wild-type l-AAD, and the *V*_max_/*K*_m_ values changed (see Table 2), suggesting that the catalytic efficiency was influenced by the affinity of the mutants.

Finally, the combination of the two mutations selected in this study further enhanced the bioconversion ratio to 91.4%, which is 20.3% more than that of the wild-type l-AAD–containing biocatalyst (see Fig. 4A). The double mutations decreased the *K*_m_ value from 305±2.31 to 238±1.08 mM and increased the *V*_max_ from 0.91±0.01 to 1.14±0.04 mM/g per hour. As a result, the engineered l-AAD containing the whole-cell biocatalyst produced results superior to those of the wild type, with a volumetric production level of 63.6 gram/L within 24 h (Fig. 4B).

### Immobilization of the whole-cell biocatalyst

**Figure 5:**
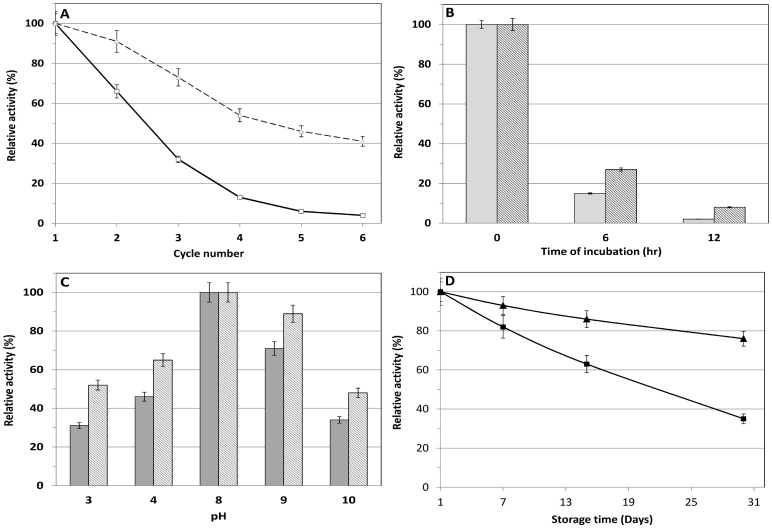
Reusability, thermal and pH stability of the immobilized whole cell biocatalyst. (A) effect of the immobilization on the reusability of the whole-cell biocatalyst [straight line with square mark (▪) for free cell and dot line with triangle mark (▴) for immobilized cell]; comparison of the thermal (B), and pH (C) (gray bar for free and crossed bar for immobilized recombinant whole cell biocatalyst); (D) storage stability between the free (square mark, ▪) and immobilized biocatalyst (triangle mark, ▴). All reactions were carried out in 20 mL of 50 mM Tris-HCl buffer (pH 8.0) at 40°C with 220 rpm for 20 h except the temperature stability experiment which was tested at 50°C for 12 h. The highest activity of the freshly prepared immobilized cell containing engineered l-AAD biocatalyst was defined as 100%.

We next determined how immobilization agents affected the production and reusability of recombinant *E. coli*. The results showed that Ca^2+^ alginate performed best among the immobilization agents (Table 3). The immobilized cell form showed superior performance during six rounds of biotransformation (Fig. 5A), including higher thermal and pH stability (Fig. 5B and 5C). Reusability was increased approximately 41.3% compared to that of the free cell (see Fig. 5A). This result suggests that l-AAD is more stable in immobilized whole cells than in free cells. The microenvironment created by the networks of the Ca^2+^ alginate bead gel may shield the biocatalyst from the effects of H^+^ ions, effectively increasing the pH stability of the biocatalyst (see Fig. 5C). Cell immobilization is a common technique for increasing the reuse and stability of biocatalysts. In addition, the separation of cells and products is much easier with immobilized cells than with free cells in suspension. Moreover, cell immobilization increase storage stability (Fig. 5D) of the biocatalyst and allow continuous biotransformation.

**Table 3:** Catalytic performance of engineered double mutant l-AAD containing *E. coli* cells immobilized in various matrices.

Immobilizing matrix	Catalyst load (g-dw/g)	Production rate (g/L^−1^h^−1^)	Yield (%)
None (free cells)	N/A ^a^	3.10±0.05	91.45
Agar	5.20±0.36	2.90±0.07	82. 17
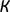 -Carrageenan	5.40±0.14	2.60±0.03	80. 21
Ca-alginate	6.30±0.21	2.90±0.09	90. 17

Conditions: 20 mL Tris-HCl buffer (50 mM, pH 8.0), with optimum amount of free cells or immobilized cells, T = 40°C. ^a^ N/A, not applicable.

## Conclusions

A powerful engineered, immobilized biocatalyst was constructed for the production of KMTB using a whole-cell biocatalyst expressing l-AAD from *P. vulgaris*. The membrane surface localization of this deaminase gene created a membrane barrier from other metabolic reactions in this process. Because the expensive cofactor FAD is omitted from the whole-cell biocatalytic procedure, our process is highly attractive in terms of expediency, productivity, and economy. Moreover, because keto acids from other amino acids are currently gaining increasing interest, we believe that our method will be useful in the production of similar keto acids, especially those from branched chain amino acids, and can be considered a keto acid production platform. Using this catalyst, we developed an efficient process for the one-step synthesis of keto acids, improving on the multi-step chemical process. The molar conversion ratio of l-methionine was 91.4%. In addition, our results showed that immobilization of the whole-cell biocatalyst enhanced the reusability of the system. Thus, the key challenge is to refine the transformation process to the point at which biotransformation competes with chemical synthesis for the production of KMTB.

## Supporting Information

S1 Fig **Comparison the KMTB production rate by two l-AAD (one from** ***P. mirabilis*** **, pm1 and another from** ***P. vulgaris*** **, pvLAAD).**
(TIF)

S2 Fig **Kinetic parameters data by Eadie-Hofstee plot and fittings.** (A) for LAAD/*E. coli* BL21 (DE3); (B) for Lys104Arg/*E. coli* BL21 (DE3); (C) for Ala337Ser/*E. coli* BL21 (DE3); (D) for Lys104Arg.Ala337Ser/*E. coli* BL21 (DE3).
(TIF)
