# Supplementary figures and images for: One-Step Biosynthesis of α-Keto-γ-Methylthiobutyric Acid from L-Methionine by an Escherichia coli Whole-Cell Biocatalyst Expressing an Engineered L-Amino Acid Deaminase from Proteus vulgaris
Source: PLoS One. 2014 Dec 22;9(12):e114291. doi: 10.1371/journal.pone.0114291 (PMC4273966; doi:10.1371/journal.pone.0114291)

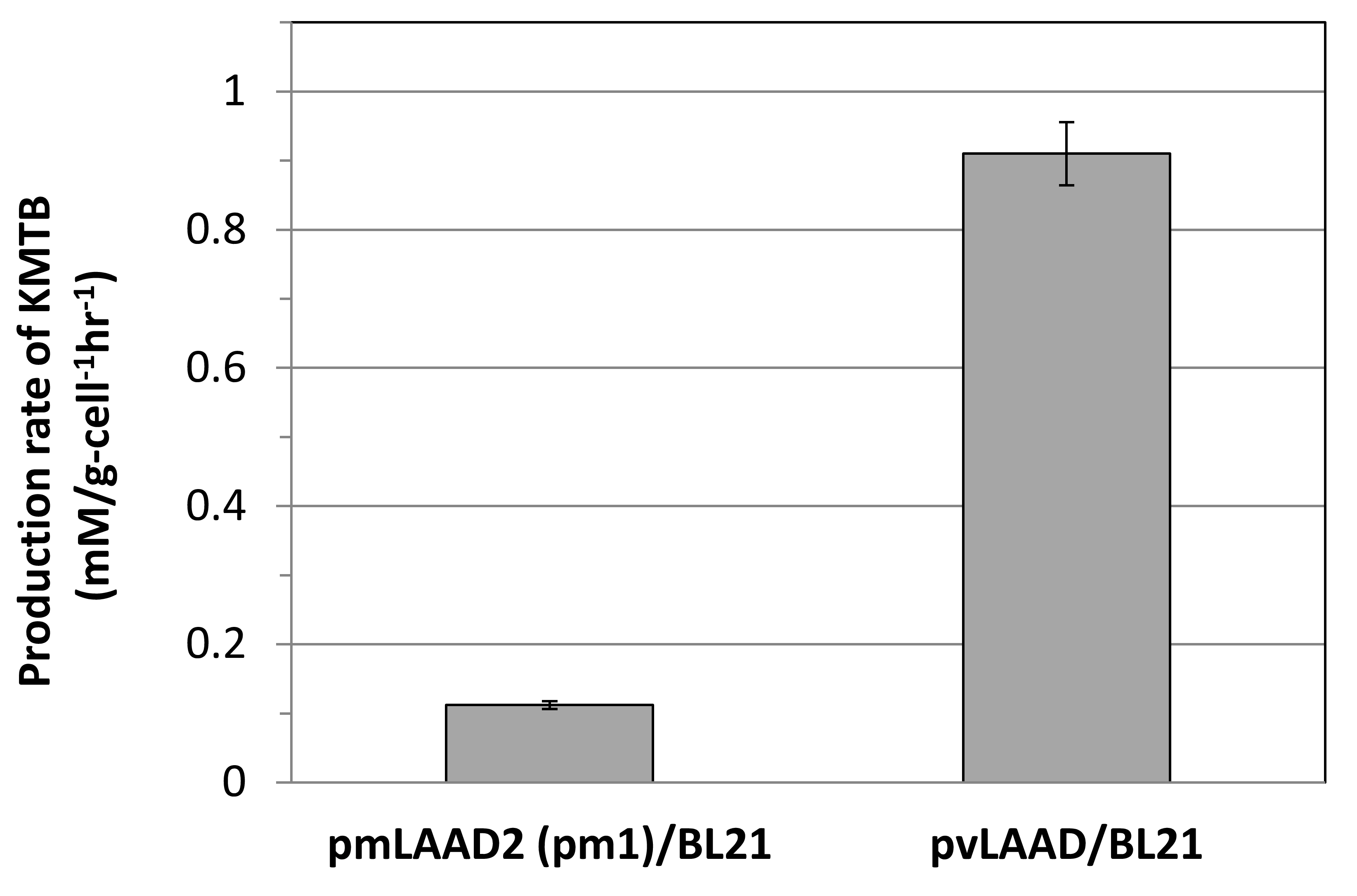

Supplement: S1 Fig — Comparison the KMTB production rate by two l-AAD (one from P. mirabilis , pm1 and another from P. vulgaris , pvLAAD). (TIF) [file pone.0114291.s001.tif]

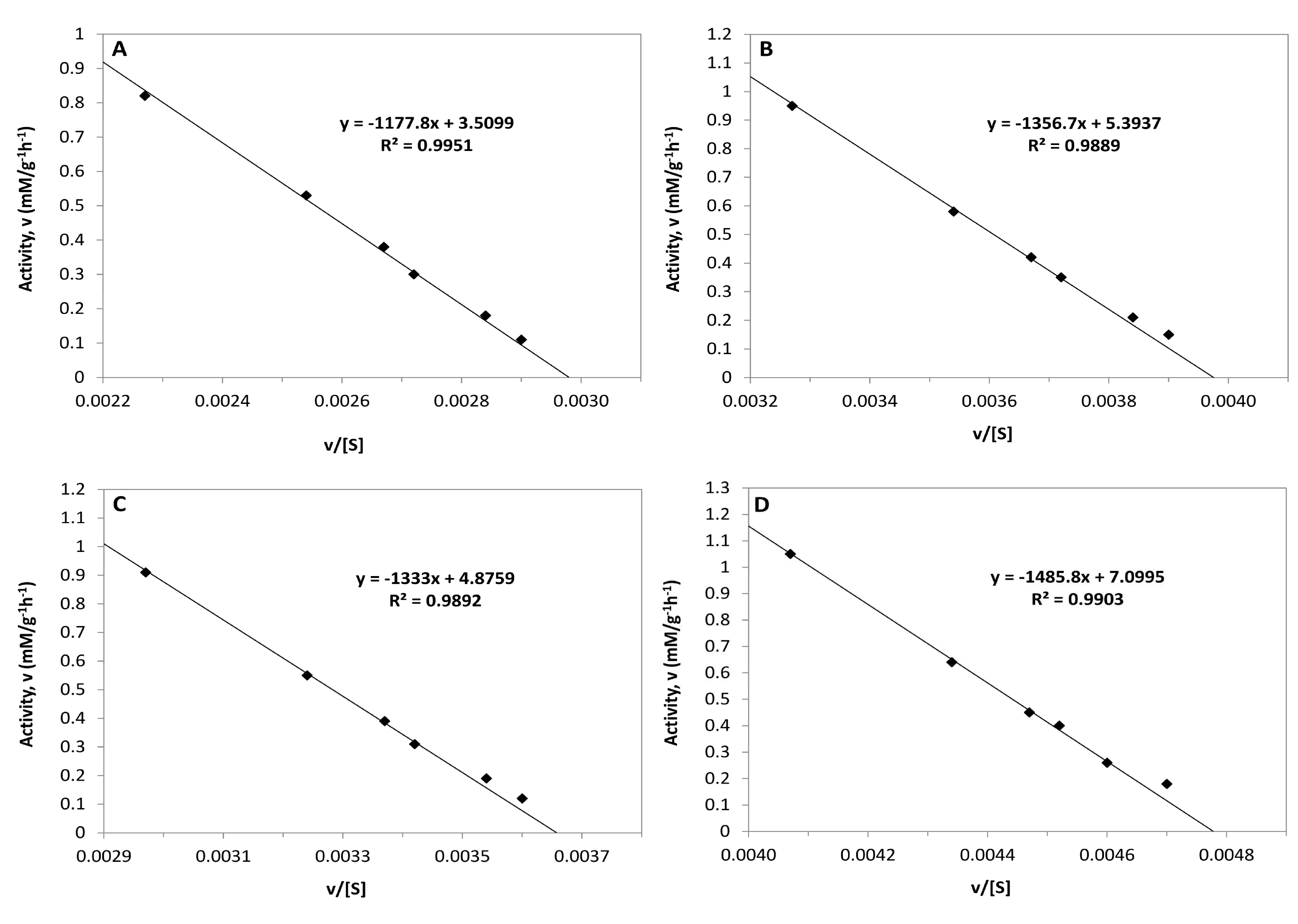

Supplement: S2 Fig — Kinetic parameters data by Eadie-Hofstee plot and fittings. (A) for LAAD/E. coli BL21 (DE3); (B) for Lys104Arg/E. coli BL21 (DE3); (C) for Ala337Ser/E. coli BL21 (DE3); (D) for Lys104Arg.Ala337Ser/E. coli BL21 (DE3). (TIF) [file pone.0114291.s002.tif]
